# Pollinator‐Promoting Interventions in European Urban Habitats—A Synthesis

**DOI:** 10.1111/ele.70189

**Published:** 2025-08-18

**Authors:** Gabriella Süle, András Báldi, David Kleijn, Ingolf Steffan‐Dewenter, Stephen Venn, Dave Goulson, Simon Dietzel, Audrey Muratet, Lorna J. Cole, Erik Öckinger, Olga Tzortzakaki, Weronika Banaszak‐Cibicka, Oliver Betz, Lorna M. Blackmore, Łukasz Dylewski, Benoît Fontaine, Bertrand Fournier, Costanza Geppert, Janine Griffiths‐Lee, Catriona Hawthorn, Andrea Holzschuh, Jakub Horák, Svenja Horstmann, Helen Hoyle, Vassiliki Kati, Anikó Kovács‐Hostyánszki, Lorenzo Marini, Alice Michelot‐Antalik, Marco Moretti, Briony A. Norton, Benjamin B. Phillips, Milan Plećaš, Patrik Rada, Miklós Sárospataki, Sonja Schulze, Assaf Shwartz, Philipp Unterweger, Viktor Szigeti

**Affiliations:** ^1^ Lendület Ecosystem Services Research Group Institute of Ecology and Botany, HUN‐REN Centre for Ecological Research Vácrátót Hungary; ^2^ Plant Ecology and Nature Conservation Group Wageningen University Wageningen the Netherlands; ^3^ Department of Animal Ecology and Tropical Ecology, Biocenter University of Würzburg Würzburg Germany; ^4^ Department of Invertebrate Zoology & Hydrobiology, Faculty of Biology & Environmental Protection University of Lodz Lodz Poland; ^5^ Department of Ecology & Evolution, School of Life Sciences University of Sussex Brighton UK; ^6^ Chair of Restoration Ecology, TUM School of Life Sciences Technical University of Munich Freising Germany; ^7^ Laboratoire Image Ville Environnement (LIVE), CNRS UMR 7362 University of Strasbourg Strasbourg France; ^8^ Integrated Land Management Scotland's Rural College Ayr UK; ^9^ Department of Ecology Swedish University of Agricultural Sciences Uppsala Sweden; ^10^ Department of Biology University of Patras Patras Greece; ^11^ Department of Zoology Poznań University of Life Sciences Poznań Poland; ^12^ Institute of Evolution and Ecology, Evolutionary Biology of Invertebrates University of Tübingen Tübingen Germany; ^13^ School of Biological Sciences University of East Anglia Norwich UK; ^14^ Patrinat & UMR7204 (CESCO), OFB‐MNHN‐CNRS‐IRD Paris France; ^15^ Institute of Environmental Science and Geography University of Potsdam Potsdam Germany; ^16^ Department of Agronomy, Food, Natural Resources, Animals and Environment University of Padova Legnaro Italy; ^17^ Faculty of Science University of Hradec Králové Hradec Králové Czech Republic; ^18^ Faculty of Forestry and Wood Sciences Czech University of Life Sciences Prague Prague Czech Republic; ^19^ Agricultural Landscapes and Biodiversity Agroscope Zürich Switzerland; ^20^ School of Architecture and Landscape University of Sheffield Sheffield UK; ^21^ Biodiversity Conservation Laboratory, Department of Biological Applications and Technology University of Ioannina Ioannina Greece; ^22^ INRAE, Lae University of Lorraine Nancy France; ^23^ Swiss Federal Research Institute WSL Biodiversity and Conservation Biology Birmensdorf Switzerland; ^24^ Nature‐Based Solutions Research Centre University of Derby Derby UK; ^25^ Environment and Sustainability Institute University of Exeter Penryn UK; ^26^ Faculty of Biology University of Belgrade Belgrade Serbia; ^27^ Department of Zoology and Ecology, Institute of Wildlife Management and Nature Conservation Hungarian University of Agriculture and Life Sciences Gödöllő Hungary; ^28^ Human and Biodiversity Research Group, Faculty of Architecture and Town Planning Technion‐Israel Institute of Technology Haifa Israel; ^29^ Dr. Unterweger Biodiversitätsplanung Wain Germany

**Keywords:** city, conservation, data synthesis, extensive mowing, flower sowing, green infrastructure, meta‐analysis, pollinator‐promoting interventions, restoration measures, urban areas

## Abstract

Pollinators receive considerable interest due to their fundamental role in ecosystem functioning and human well‐being. Unlike farmlands, studies of urban pollinator‐promoting interventions are scarce and have not been synthesised, hampering policy implementation. To fill this gap, we compared pollinator‐promoting interventions (treatment) with conventionally managed (control) sites regarding vegetation, floral resources, and pollinators. Our synthesis investigated 1051 sampling sites with different interventions (abandonment, extensive mowing, flower sowing, and combined practices) and habitats (parks, grasslands, road verges, private and public gardens) from 28 European datasets at pooled‐ and study‐levels. Urban pollinator‐promoting interventions generally benefited plants and pollinators with taxon, intervention, habitat, and spatio‐temporal specific differences. Pooled analyses showed mostly positive and never negative treatment effects, while study‐level details described primarily positive and neutral but rarely negative effects. Bumblebees and butterflies benefited most from the interventions. Some effects were stronger for interventions involving flower sowing, interventions occurring in road verges, and interventions located in Northwestern Europe. Although regulations, guidelines, and monitoring are improving, knowledge gaps remain for some pollinator taxa (e.g., beetles), regions (e.g., Mediterranean), and novel interventions (e.g., for ground‐nesting insects). Further collaborative studies from around the world could help cities bring people, plants, and pollinators together by creating resilient, multi‐functional urban spaces.

## Graphical abstract

Syntheses of urban pollinator‐friendly management are lacking, hindering policy implementation compared to farmland. Addressing this gap, our re‐analytical data synthesis demonstrates the generally positive impacts of urban pollinator‐promoting interventions (treatment) compared to conventional management (control). Further broad collaborations could bridge knowledge gaps on taxa, regions, and novel interventions to improve the urban coexistence of people, plants, and pollinators.

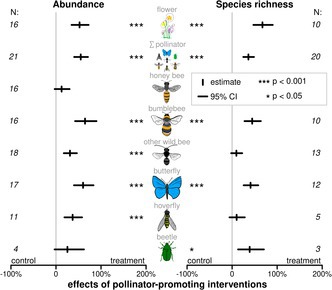

## 1 Introduction

By facilitating plant reproduction, pollinators play a fundamental role in supporting healthy terrestrial ecosystems and sustainable food production (IPBES 2016). However, worldwide pollinator abundance and diversity are declining due to land use intensification, climate change, pesticide use, and diseases (Goulson et al. 2015; Ollerton 2017; Potts, Biesmeijer, et al. 2010). The combination of their key role in maintaining populations of both wild and cultivated plants, alongside their evident negative population trends (Potts, Biesmeijer, et al. 2010), identifies pollinators as a flagship group for nature conservation (Kovács‐Hostyánszki et al. 2017; Meldrum et al. 2023). Consequently, there is an ever‐increasing number of studies investigating their ecology, population trends, and especially the effectiveness of management interventions to conserve them. Most of these studies focus on interventions promoting pollinators in agricultural landscapes, where there is serious concern about the consequences of the loss of pollination functions for productivity and food security (Corbet et al. 1991; Vanbergen et al. 2020). Agri‐environmental schemes in Europe and North America offer financial support for the implementation of pollinator‐friendly practices on agricultural land (Gohin and Zheng 2020). These schemes create a standardised framework that facilitates empirical studies of such practices in real‐world settings (Boetzl et al. 2021). As a result, now we have a good understanding of the ecological effectiveness of many pollinator‐promoting interventions within agricultural settings (Kovács‐Hostyánszki et al. 2017), including flower strips (Albrecht et al. 2020), field margins (Marshall 2005), set‐asides (Kovács‐Hostyánszki et al. 2021), and organic farming (Carrié et al. 2018).

Pollinators such as butterflies and bumblebees are charismatic species groups for the general public (Guiney and Oberhauser 2008; Skaldina and Blande 2025), leading to the increasing implementation of pollinator‐promoting interventions in villages and cities (Baldock 2020). More than half of the human population lives in cities (Potter 2013), which currently cover around 2% of land globally, and both of these statistics are continuously increasing (Seto et al. 2012; Taubenböck et al. 2024). The main focus of ecological studies on urban pollinators has centred on how communities differ from agricultural habitats (Baldock et al. 2015; Theodorou et al. 2017) and the adverse effects of habitat loss due to urbanisation (Banaszak‐Cibicka and Żmihorski 2020; Liang et al. 2023; Persson et al. 2020; Prendergast et al. 2022). However, in comparison with intensively managed agricultural land, urban landscapes (including parks, road verges, and gardens) can provide refuge for pollinators (Hall et al. 2017). Pollinator‐promoting interventions can also enhance these habitats (Baldock 2020; Kleijn et al. 2020), and cities are motivated to implement strategies supporting urban biodiversity and pollinators in particular (Wilk et al. 2019). Still, the research on this topic has only emerged in the last decades (Hall et al. 2017; Norton et al. 2019; Phillips et al. 2020; Valtonen et al. 2006). Despite the growing number of studies on the efficacy of pollinator‐promoting interventions in urban areas, we still lack comprehensive overviews.

In urban environments, replacing the conventional management techniques could have positive impacts on pollinators (Baldock 2020; Horák et al. 2022; Vélová et al. 2023). For instance, short‐term abandonment or reduced mowing frequency are easy‐to‐implement and cost‐effective options in urban green spaces, comparable to agricultural set‐asides (Garbuzov et al. 2015; O'Sullivan et al. 2017). These measures allow the vegetation to grow, naturally increasing floral diversity and providing nesting resources, especially in comparison to conventional maintenance (Wastian et al. 2016). Farmlands often implement flower strips (Albrecht et al. 2020; Báldi et al. 2022); similarly, public spaces increasingly incorporate areas sown with various flowering seed mixtures for pollinators (Blackmore and Goulson 2014; Dietzel et al. 2023; Norton et al. 2019; Süle et al. 2023). In addition to providing floral resources, these habitat patches can accommodate larvae and overwintering stages, concurrently providing multiple ecosystem services, such as buffering microclimatic conditions, retaining water, and offering aesthetic value for citizens as co‐benefits (Lange‐Kabitz et al. 2021; Noordijk et al. 2009; Southon et al. 2017; Unterweger et al. 2018; Wintergerst et al. 2021). Artificial nesting sites, such as‘bee hotel’ and bare ground surfaces, promote cavity‐ and ground‐nesting species (Baldock 2020; Fortel et al. 2016; Potts et al. 2005), sometimes with unintended effects (e.g., facilitating alien species and pathogen spillover (Geslin et al. 2020; MacIvor and Packer 2015)). Furthermore, similar to agricultural systems, decreased use of herbicides, insecticides, and fertilisers has a positive effect on environmental health in cities (Muratet and Fontaine 2015; Winqvist et al. 2012). All of these pollinator‐promoting interventions could be applied not only in public spaces but also in private gardens, allotments, green roofs, and balconies (Baldock et al. 2019; Foster et al. 2017; Shwartz et al. 2014). Furthermore, implementing these initiatives in urban environments is less likely to conflict with food production, which can be a concern in agricultural settings, while more likely to provide opportunities to improve the public perception of insects, ecosystem functions, and associated services (Fukano and Soga 2021; Geppert et al. 2024).

In parallel, the involved citizens, stakeholders, municipalities, countries, and international organisations are eager to halt the decline of pollinating insects and maintain greener cities, especially in the EU (Hering et al. 2023; UN DESA 2023). Europe has experienced significant biodiversity loss (Hermoso et al. 2022), but nowadays, attention is shifting to the provision of an environment facilitating citizen health and well‐being. In the case of pollinators, the development and restoration goals of the public and decision‐makers seem to coincide (Council of the EU 2023; European Parliament 2024). However, the efficiency of the interventions may differ regionally due to socio‐economic differences and ecological conditions within Europe (Batáry et al. 2010; Kronenberg 2015; Southon et al. 2017). Urban habitats also vary in their types and management. For instance, when comparing Nordic and Mediterranean cities, a park or road verge requires different mowing schedules and will be affected differently by abandonment due to succession processes or even invasion (Chytrý et al. 2009; Horstmann et al. 2024; Öckinger et al. 2009; Tzortzakaki et al. 2019). Similarly, temporal attributes could also influence impacts because interventions do not always manifest immediately; their benefits may peak and decline over years after implementation (Buhk et al. 2018; Pywell et al. 2011). Although stakeholders' decisions could draw from studies of farmland pollinators, evidence from urban pollinator‐promoting interventions is urgently needed to inform best practice (Tremblay and Underwood 2023; Wilk et al. 2019). Even though case studies and reviews are widely available (Baldock 2020; Braman and Griffin 2022; Glenny et al. 2022; O'Sullivan et al. 2017; Phillips et al. 2020), a comprehensive quantitative synthesis, such as a meta‐analysis or re‐analytical data synthesis, is lacking for urban pollinator‐promoting interventions (but see the synthesis of Millard et al. (2021) on land‐use intensity).

In this synthesis, we addressed this knowledge gap by reviewing the effectiveness of urban pollinator‐promoting interventions across Europe. We carried out a re‐analytical data synthesis based on 28 primary datasets from 12 European countries. In contrast to extracting data from publications for a meta‐analysis, gathering datasets for re‐analytical data synthesis is more labour‐intensive (Tudur Smith et al. 2016). Classical meta‐analysis combines and analyses the results of multiple independent studies (Gurevitch et al. 2018). While re‐analytical data synthesis collects and combines raw data from multiple studies, providing a more detailed understanding by offering the opportunity of generating robust models that incorporate study‐level variances, site‐level, and seasonal factors, it uses the original data (Riley et al. 2023). This approach also increases engagement with the researchers who originally collected the data, as they can provide published and unpublished datasets with high resolution, including the pollinator taxa, spatio‐temporal details, and site‐level background variables. Data owners can also contribute better insight into any nuances detected and findings highlighted. However, a re‐analytical data synthesis may suffer from not including all the existing datasets due to e.g., data owners lacking time for any extra task, receiving too many requests with few successful publication outcomes, and even mistrusting less‐known researchers (Renzl 2008; Stieglitz et al. 2020). We have carried out the first data‐based synthesis on urban pollinator‐promoting interventions in Europe, while a global meta‐analysis still remains to be implemented.

Our main research aim was to determine whether pollinator‐promoting interventions have positive effects on vegetation, floral resources, and a broad range of pollinator groups. Primarily, we investigated the pooled and study‐level differences between treatment (pollinator‐promoting interventions) and control (conventional management/not intended to benefit pollinators) sites. Over the general impacts, we also investigated the potential influence of (a) intervention type (i.e., abandonment, extensive mowing, flower sowing, and combined practices), (b) habitat type (i.e., parks, grasslands, road verges, private and public gardens), (c) years after establishment (i.e., age of intervention), and (d) spatial location on the effects of interventions. Building on our wide‐scale datasets and collective expertise in pollinator ecology, we briefly highlight knowledge gaps and identify clear actions to make urban environments more favourable for pollinators while also considering wider socio‐ecological aspects.

## 2 Methods

### 2.1 Data Query and Selection Criteria

To carry out a re‐analytical data synthesis on urban pollinator‐promoting interventions, we integrated available datasets from Europe. We focused on interventions that aimed to enhance resources for pollinators, e.g., by replacing conventional green space management, reducing chemical and mechanical treatments, or sowing flowers. To find relevant studies and authors, we searched the Web of Science database (by ‘TS=topic’ tag) for publications, using three sets of search strings: (i) habitat management: ‘conservation management’, ‘conservation measure’, ‘cut*’, ‘cutting’, ‘establish*’, ‘floral addition*’, ‘flower addition*’, ‘graze*’, ‘grazing’, ‘habitat restoration’, ‘maintain*’, ‘management’, ‘mow*’, ‘mowing’, ‘planting*’, ‘pollinator conservation’, ‘pollinator friendly’, ‘promote*’, ‘restorat*’, ‘seeding*’, ‘shear*’, ‘shearing’, ‘sow*’; (ii) pollinators: ‘bee flies’, ‘bee fly’, ‘bees’, ‘butterfl*’, ‘flower visitor*’, ‘hover flies’, ‘hoverfl*’, ‘hoverfly’, ‘hymenoptera*’, ‘lepidoptera*’, ‘moth*’, ‘pollinat*’, ‘syrphid*’, ‘wasp*’; (iii) urban habitats: ‘bee forage*’, ‘bee pasture*’, ‘green space*’, ‘greenspace*’, ‘lawn*’, ‘park*’, ‘public space’, ‘road verge*’, ‘roadside verge*’, ‘urban’, ‘urban space*’. We used the ‘AND’ operator between groups and ‘OR’ between terms within groups; ‘*’ denotes wildcards. By the ‘NOT’ operator, we excluded ‘mother*’; and ‘parking’, ‘national park*’ from the second and third groups, respectively, to avoid irrelevant publications. This search on 8 March 2023 yielded 1644 publications.

We selected publications based on their titles, abstracts, and full texts by the following inclusion criteria: (1) sampled abundance and species richness of pollinators; (2) examined habitats within urban areas; (3) were conducted in Europe; (4) included different management intensities; (5) at least three treatment sites (pollinator‐promoting management) and three control sites (conventional management without aims on pollinators). We excluded papers that studied only non‐pollinating forms of insects (e.g., larval stages). To reach authors with suitable datasets (only one data was openly available (Norton et al. 2019)), we contacted the corresponding authors of all 44 selected papers. Furthermore, to access non‐published data, we also contacted colleagues working on this topic. We sent out data‐gathering emails to 77 potential data owners in several repeated rounds between 1 October 2022 and 1 May 2023. Of the 77 data owners contacted, 33 responded; 11 lacked suitable datasets for our synthesis, and 3 reported datasets overlapping with those already received from other data owners. Finally, we gathered 28 datasets from 12 European countries fitting the scope of this study.

### 2.2 Datasets

We requested information on the study design (e.g., intervention and habitat types), data on the average vegetation height, percentage of vegetation coverage (only the green parts of plants; hereafter: ‘vegetation cover’), and the abundance and species richness of floral resources and pollinator groups (i.e., honey bee, bumblebee, other wild bee, butterfly, hoverfly, other fly, and flower‐visiting beetle; see details in Table S1), together with site coordinates (projection: EPSG:4326‐WGS84). For four datasets, the exact site coordinates were not available; here we used the central coordinates of the sampled region or city or the same coordinates for site pairs. We gathered data at the site level and at the highest possible resolution (e.g., without pooling sampling periods), including information on sampling dates and periods. Vegetation parameters were gathered as a proxy for the intensity of green space management, availability of floral and further plant‐related resources for pollinators (Dylewski et al. 2020; Klein et al. 2020; Milberg et al. 2016). We standardised and merged the (names of) variables for analyses (see details in Table S1). Thus, ‘flower abundance’, besides the number of flower units, also included flower cover (five cases) and categorical flowering intensity (four cases). The category of ‘other wild bee’ included all bees in one case and all Hymenoptera except Formicidae in two cases; ‘butterfly’ included all Lepidoptera in one case; ‘flower‐visiting beetle’ included all Coleoptera (Table S1). These variables were always uniform within primary datasets. Furthermore, we calculated the total abundance and species richness for all pollinators where > 1 pollinator group was sampled.

**FIGURE 1:**
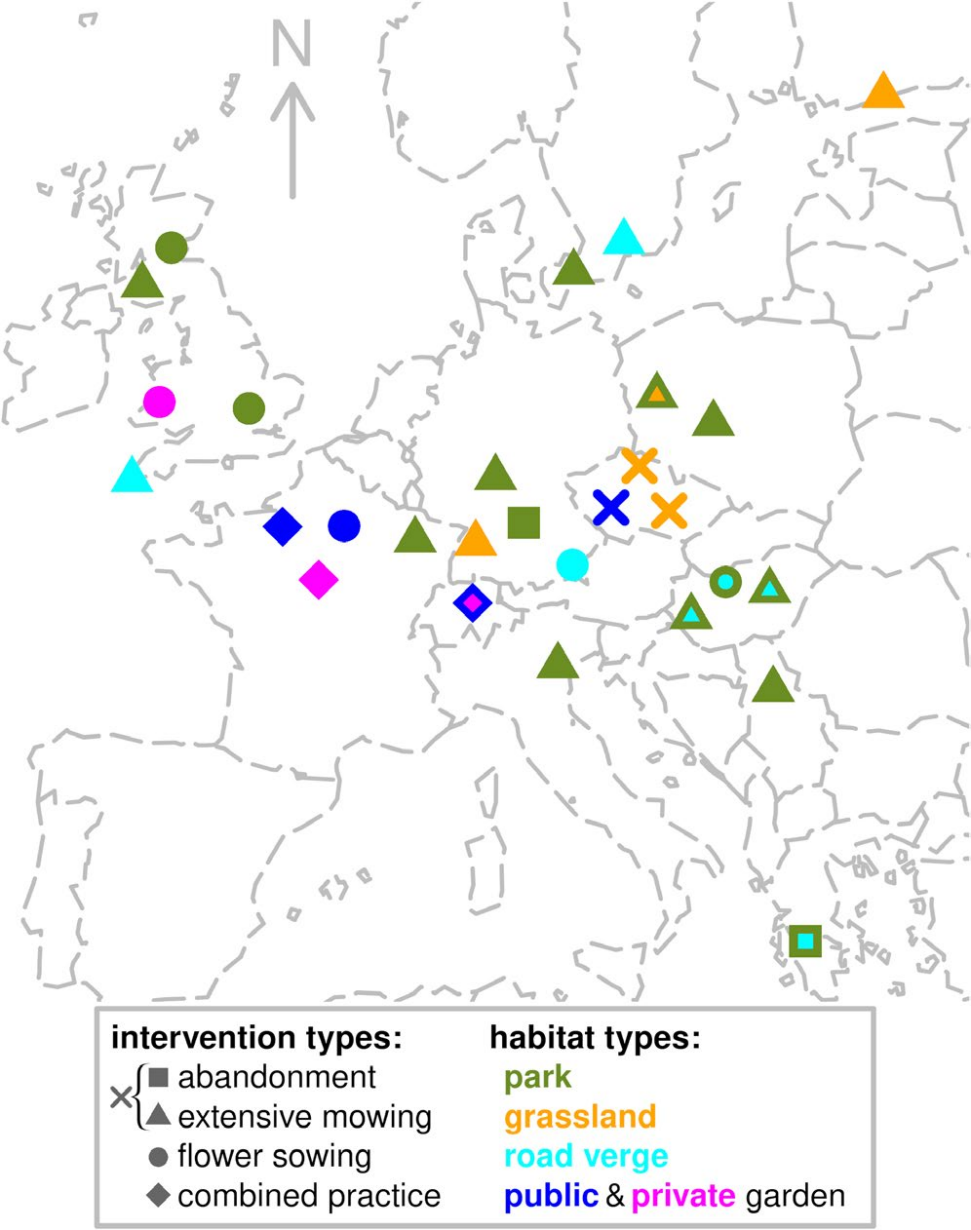
The gathered datasets within Europe. Coloured points present the involved studies with their averaged site coordinates, slightly jittered for visualisation. Intervention types are presented by square (abandonment); triangle (extensive mowing); cross (abandonment or extensive mowing); circle (flower sowing); rhombus (combined practices). Habitat types are presented by olive green (parks); orange (urban grasslands); cyan (road verges); dark blue (public gardens); and magenta (private gardens) colours. Double colours mean more than one habitat type in a dataset. EuroGeographics for the administrative boundaries.

Altogether, we compiled primary datasets investigating the effect of pollinator‐promoting interventions on vegetation height (6 datasets), vegetation cover (5), flower abundance (abu: 16) and species richness (sp: 10), honey bee (abu: 16), bumblebee (abu: 16, sp.: 10), other wild bee (abu: 18, sp.: 13), butterfly (abu: 17, sp.: 12), hoverfly (abu: 11, sp.: 5), other fly (abu: 4, sp.: 2), flower‐visiting beetle (abu: 4, sp.: 3), and total pollinator (abu: 21, sp.: 20; see Figure 1, Table S1). Datasets cover 15 years (2006–2022) and 12 countries, with altogether 1051 sampling sites within Europe (Figure 1, Table S1). The investigated pollinator‐promoting interventions were abandonment and extensive mowing (19 primary datasets, of which three contained both intervention types), sowing of flowering plants (6), or combined practices targeting higher biodiversity (3; Figure 1, Table S1). About half of the study designs were originally control‐treatment approaches (discrete management), while the other half were categorised for this study based on expert decisions or treatment gradients (e.g., continuous management intensity differences among gardens or sites within cities; Table S1). The habitat types covered parks (including urban green spaces), urban grasslands (including ruderal habitats and lawns), road verges in cities and suburbs, as well as private and public gardens (including orchards; Figure 1, Table S1).

### 2.3 Statistical Analyses

The primary datasets were sampled with different field methods, causing inherent variation in the response variables (i.e., vegetation height and cover, flower and pollinator abundances and species richness). To analyse these on a comparable level, we scaled all values from 0 to 1 at the level of sampling periods for each response variable. Values closer to 1 indicate the most, whereas values closer to 0 indicate the least favourable sites regarding vegetation, floral resources, and pollinators (e.g., 1 means most pollinators, while 0 means the least pollinators). We scaled and analysed the following response variables (19) separately: vegetation height, vegetation cover, flower abundance, flower species richness, pollinator abundance, and pollinator species richness (8 and 7) at group level, respectively. We applied generalised linear mixed models (GLMM; (Venables and Ripley 2002; Zuur et al. 2009)) with the'ordbet' family, which handles the 0–1 values allowing lower and upper bounds (Kubinec 2023). The potential inconsistency among studies, variances over the sampled years, and pseudoreplication within studies were treated as random factors (see details below). First, to reveal the general effects of pollinator‐promoting interventions, we combined the primary datasets and analysed the response variables separately (pooled analyses). Pollinator‐promoting intervention (treatment vs. control sites) was the explanatory variable (i.e., treatment effect), while separate primary datasets and sampled sites (1|study/site), as well as sampled years and sampling periods (1|year/period), were treated as nested random factors. Second, to reveal the specific differences in treatment effect between primary datasets, we fitted similar models, including the treatment effect for each primary dataset, while incorporating them into one model, improving standard error estimates and avoiding increased Type I errors due to multiple testing (study‐level analyses). Explanatory variables were the primary datasets with reference to zero and the interaction between datasets and pollinator‐promoting intervention (i.e., ~0 + study+study:treatment). The sites, years, and sampling periods were random factors.

To investigate the influence of (a) intervention type, (b) habitat type, (c) years after establishment, and (d) spatial location, in addition to the control‐treatment effects on the response variables (i.e., vegetation height and cover, flower and pollinator abundance and species richness), we applied AIC‐based model comparison between our original pooled models on general treatment effects (basic model) and models including these additional (a–d) factors (full model), separately. We ran full models for each response variable where > 5 primary datasets were available. To reveal the influencing role of (a) intervention and (b) habitat type on the response variables, the full model included intervention type or habitat type and their interactions with treatment as additional factors, separately. Due to the low number of studies within specific types of intervention (abandonment) and habitat (urban grassland, private and public garden), group merging was necessary. Thus, we were able to analyse the impact and differences among (a) interventions (abandonment and extensive mowing (as a pooled group), flower sowing, combined practice) and (b) habitats (park and urban grassland (pooled), road verge, private and public garden (pooled)).

To reveal the impact of (c) years after establishment on the response variables, analyses were restricted to the subset of primary datasets where this information was available. We compared the basic model (run on the subset of datasets) with a more complex model that also included years after establishment and its interaction with treatment. Additionally, to reveal non‐linear time effects, a further model was compared that also included the second‐order polynomials of years after establishment. To reveal the influence of (d) spatial location on the response variables, full models included the coordinates of the sampling sites as structured spatial exponential covariance matrices grouped by treatment (Kristensen et al. 2016; Kristensen and McGillycuddy 2024). The random terms were always kept the same as in the original basic models.

We compared the AIC values of the basic and full models separately for each variable. If the full model has a lower AIC (ΔAIC > 2), it indicates that the extra (a–d) factors influenced the effect of pollinator‐promoting interventions. For full models with lower AICs, we present model estimates on treatment effect differences between (a) intervention types and (b) habitat types, as well as predictions on (d) spatial patterns of the control‐treatment difference. Note: we used the original values for 0–1 scaling in all response variables, with the exception of honey bees. The rare extreme abundances of honey bees, as a single species heavily reliant on beekeeping management, posed challenges to the convergence of the study‐level model. To handle this, we calculated the square root of abundances before 0–1 scaling. We checked that the pooled models produce similar results and that any of the significant effects change if we use the original scaled values for honey bees. We used the R statistical environment (v.4.2.1; RCoreTeam 2025, packages ‘glmmTMB’ v.1.1.5 for GLMMs; Brooks et al. 2017, and ‘sf’ v.1.0‐14 for spatial layers; Pebesma 2018).

## 3 Results

Gathered datasets on urban pollinator‐promoting interventions integrated 28 primary field studies from 360 settlements in 12 European countries (Figure 1, Table S1). The datasets covered 1–3 years, 1–7 pollinator groups, and measured the impacts of abandonment, extensive mowing, flower sowing, and combined practices at 6–291 sites in parks (27.9% of sites across all primary datasets), grasslands (13.4%), road verges (12.7%), public and private gardens (46.1%; Figure 1, Table S1).

**FIGURE 2:**
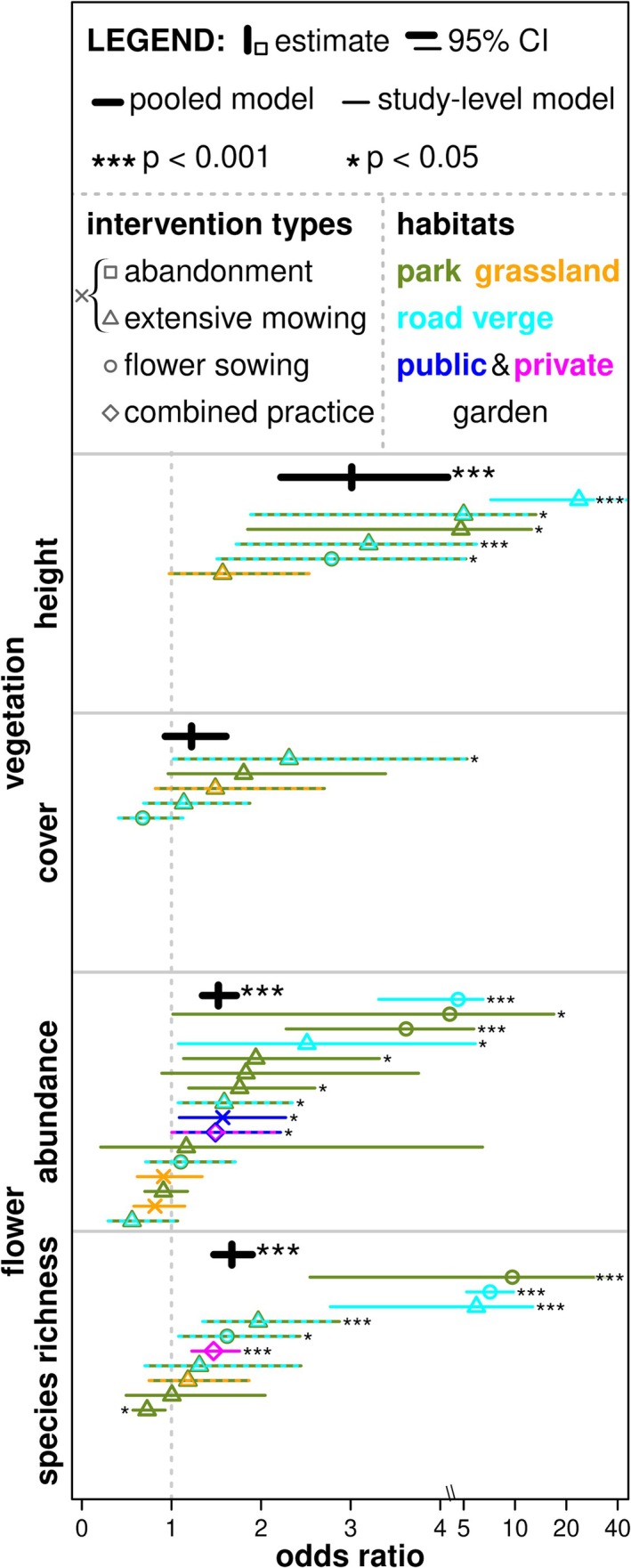
Impacts of pollinator‐promoting interventions on vegetation height, vegetation cover, flower abundance and species richness. Odds ratio is the exponential of the model estimates and 95% confidence intervals. A value above one means a positive impact of urban pollinator‐promoting interventions (treatment) compared to conventional management (control). The pooled models are presented with thick black lines, and stars indicate significant positive differences (***p < 0.001). Study‐level models are presented with thinner, colourful lines with symbols, where stars also indicate significant differences (*p < 0.05 and ***p < 0.001) on the corresponding side (as positive or negative effect) of the lines. Intervention types are presented by triangle (extensive mowing); cross (abandonment or extensive mowing); circle (flower sowing); rhombus (combined practices). Habitat types are presented by olive green (parks); orange (urban grasslands); cyan (road verges); dark blue (public gardens); magenta (private gardens) colours; dashed lines and double colours mean more than one habitat type in a dataset. X‐axis changes above value four to the log scale for better visualisation. Please see Tables 1 and S2 for the exact model estimates.

**FIGURE 3:**
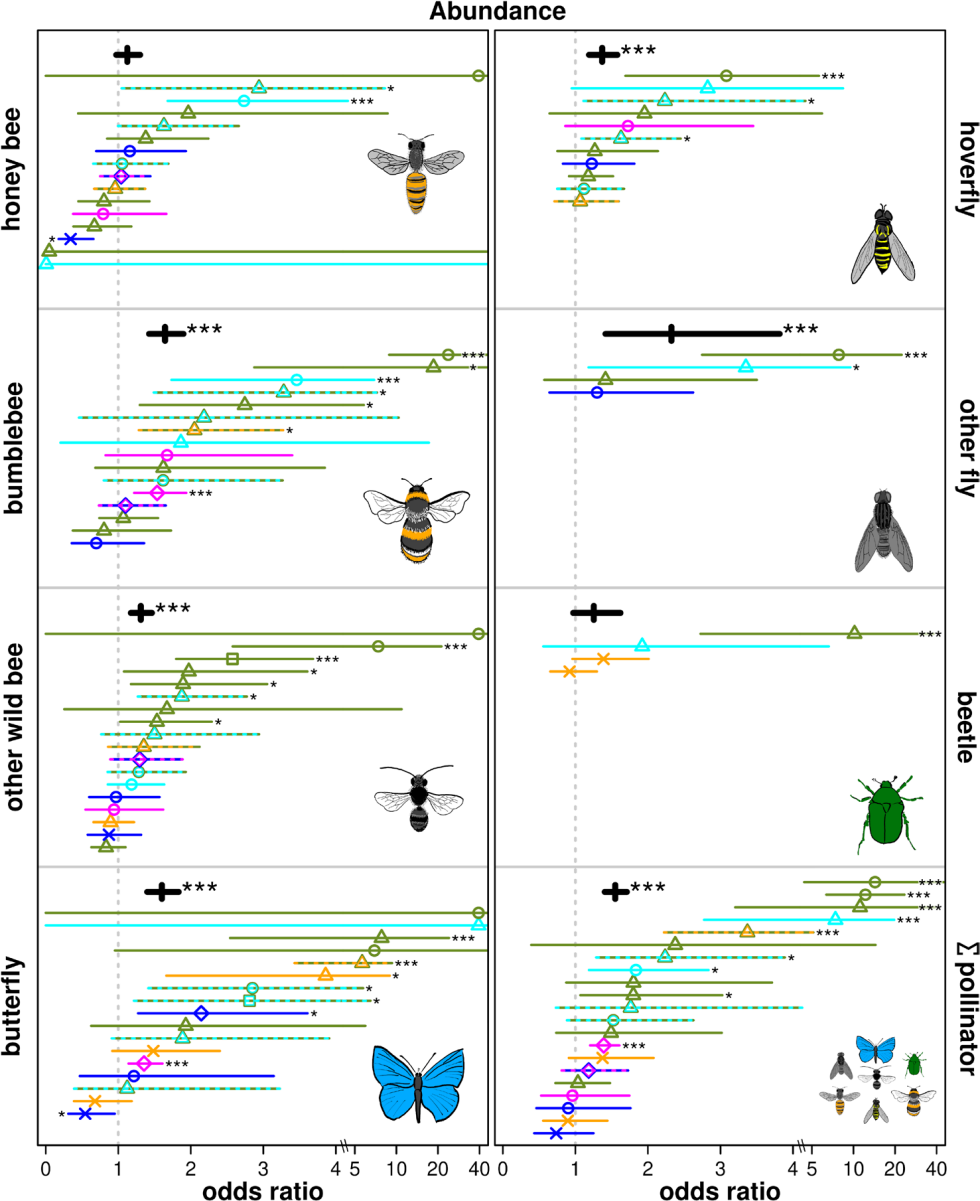
Impact of pollinator‐promoting interventions on abundance of pollinator groups. Odds ratio is the exponential of the model estimates and 95% confidence intervals. A value above one means a positive impact of urban pollinator‐promoting interventions (treatment) compared to conventional management (control). The pooled models are presented with thick black lines, and stars indicate significant positive differences (***p < 0.001). Study‐level models are presented with thinner, colourful lines with symbols, where stars also indicate significant differences (*p < 0.05 and ***p < 0.001) on the corresponding side (as positive or negative effect) of the lines. Intervention types are presented by square (abandonment); triangle (extensive mowing); cross (abandonment or extensive mowing); circle (flower sowing); rhombus (combined practices). Habitat types are presented by olive green (parks); orange (urban grasslands); cyan (road verges); dark blue (public gardens); magenta (private gardens) colours; dashed lines and double colours mean more than one habitat type in a dataset. X‐axes change above value four to the log scale for better visualisation. For honey bee, other wild bee, and butterfly abundances, four outlier estimates are presented at the value of 40. Please see Tables 1 and S2 for the exact model estimates.

**FIGURE 4:**
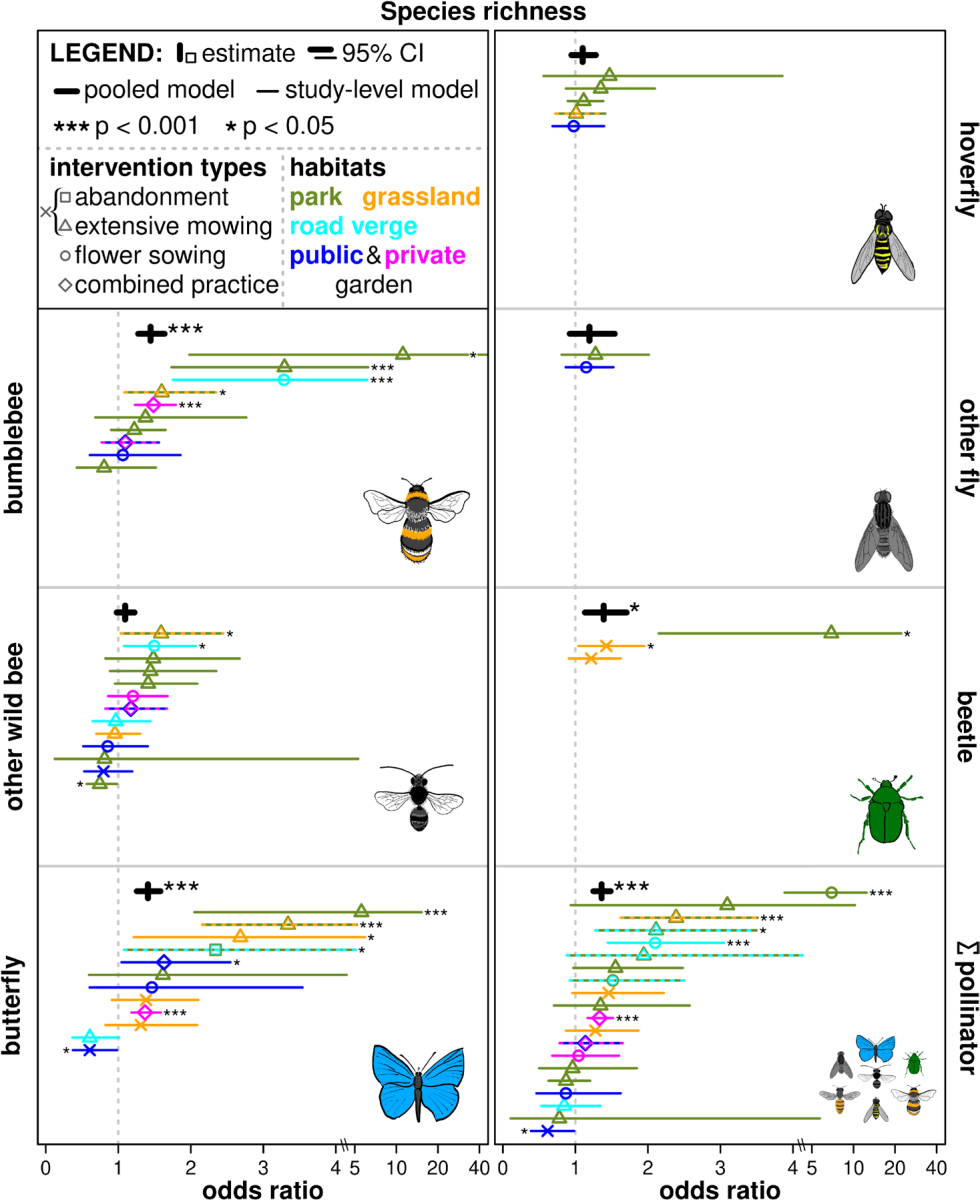
Impact of pollinator‐promoting interventions on species richness of pollinator groups. Odds ratio is the exponential of the model estimates and 95% confidence intervals. A value above one means a positive impact of urban pollinator‐promoting interventions (treatment) compared to conventional management (control). The pooled models are presented with thick black lines, and stars indicate significant positive differences (*p < 0.05 and ***p < 0.001). Study‐level models are presented with thinner, colourful lines with symbols, where stars also indicate significant differences (*p < 0.05 and ***p < 0.001) on the corresponding side (as positive or negative effect) of the lines. Intervention types are presented by square (abandonment); triangle (extensive mowing); cross (abandonment or extensive mowing); circle (flower sowing); rhombus (combined practices). Habitat types are presented by olive green (parks); orange (urban grasslands); cyan (road verges); dark blue (public gardens); magenta (private gardens) colours; dashed lines and double colours mean more than one habitat type in a dataset. X‐axes change above value four to the log scale for better visualisation. For honey bee, other wild bee, and butterfly abundances, four outlier estimates are presented at the value of 40. Please see Table 1 for the exact model estimates.

Pooled analyses on all datasets together presented significant positive effects of pollinator‐promoting interventions on vegetation height, flower abundance and species richness (Figure 2 and Table 1). There were significant positive effects from the pooled analyses on the abundance of bumblebees, other wild bees, butterflies, hoverflies, other flies, and total pollinators (Figure 3) and species richness of bumblebees, butterflies, flower‐visiting beetles, and total pollinators (Figure 4 and Table 1). The effects were never negative but neutral for vegetation cover, abundance of honey bee, and flower‐visiting beetles, as well as species richness of other wild bees, hoverflies, and other flies (Figures 2, 3, 4, Table 1). Study‐level analyses presented 37.3% of significant positive effects, while also a few (2.9%) negative effects (Figures 2, 3, 4, Table S2). High variances in studies, sites, and sampling periods as random factors suggested their importance. In contrast, sampled years had smaller random effects in most cases (Tables 1 and S2).

**TABLE 1:** Results of generalised linear mixed models (GLMMs) analysing the effects of pollinator‐promoting intervention on the vegetation height and cover, abundance and species richness of flowers and pollinator groups at the pooled level. Significant *p*‐values (< 0.05) are in bold.

Response variables		Estimate	SE	*p*	Standard deviation of random terms			
Variable	Type of value				Studies	Sites	Years	Periods
Vegetation	Height	1.102	0.155	**< 0.001**	0.137	0.706	< 0.001	0.366
	Cover	0.202	0.139	0.1480	0.534	0.529	< 0.001	0.614
Flower	Abundance	0.421	0.063	**< 0.001**	0.483	0.426	0.390	0.336
	Species richness	0.515	0.065	**< 0.001**	< 0.001	0.485	< 0.001	0.272
Honey bee	Abundance	0.117	0.074	0.1149	0.938	0.430	< 0.001	0.504
Bumblebee	Abundance	0.498	0.073	**< 0.001**	0.777	0.608	< 0.001	0.711
	Species richness	0.368	0.064	**< 0.001**	0.566	0.472	< 0.001	0.361
Other wild bee	Abundance	0.271	0.056	**< 0.001**	0.417	0.391	0.164	0.547
	Species richness	0.091	0.057	0.1107	0.023	0.380	0.214	0.363
Butterfly	Abundance	0.471	0.069	**< 0.001**	0.412	0.778	1.288	0.718
	Species richness	0.343	0.058	**< 0.001**	0.301	0.635	< 0.001	0.319
Hoverfly	Abundance	0.315	0.073	**< 0.001**	0.571	0.226	< 0.001	0.527
	Species richness	0.096	0.078	0.2182	0.237	0.204	< 0.001	0.246
Other fly	Abundance	0.843	0.253	**< 0.001**	0.282	0.674	0.282	< 0.001
	Species richness	0.177	0.131	0.1759	0.326	0.169	0.326	0.238
Flower‐visiting beetle	Abundance	0.226	0.131	0.0846	0.108	0.357	0.108	0.194
	Species richness	0.329	0.105	**0.0017**	< 0.001	0.255	< 0.001	0.178
Total pollinator	Abundance	0.438	0.050	**< 0.001**	0.481	0.624	0.064	0.454
	Species richness	0.309	0.044	**< 0.001**	0.276	0.554	0.066	0.317

Models including (a) intervention and (b) habitat types had a lower AIC than the basic models in some cases (30.8% and 69.2%, respectively; Table S3). The results of four full models with lower AICs for intervention types suggest that flower sowing had a greater impact compared to other types of interventions on flower abundance and species richness, abundance of bumblebees and total pollinators (Table S4). In the case of full models with lower AICs for habitat types, interventions in road verges had the greatest impact in most cases (i.e., on flower abundance and species richness, honey bee abundance, and bumblebee species richness; Table S5). Interventions in parks and grassland had the greatest impact in the models of butterfly species richness (Table S5). Interventions in gardens had the least impact in most models, with the exception of flower abundance and species richness models, where the impact in parks and grassland was similarly low or lower (Table S4). Models, which included (c) years after establishment, had lower AIC values in four cases (30.8%): vegetation height, flower abundance and species richness, and bumblebee abundance (Table S5). Models including (d) spatial location presented a lower AIC in most cases (84.6%; Table S3). The spatial distribution of treatment effects showed a northwest–southeast gradient and aggregations around some sampled cities (Figures S1 and S2).

Altogether, based on these extended models, including four additional factors (a–d), habitat type and spatial location seemed to be the most influential factors. However, these details require careful consideration due to the limited replications of intervention and habitat types, as well as the relatively limited spatio‐temporal coverage.

## 4 Discussion

### 4.1 Positive Effects of Urban Pollinator‐Promoting Interventions

Our data‐driven synthesis demonstrates that across Europe, flowers and pollinators generally benefit from urban pollinator‐promoting interventions. However, the details matter, as treatment effects vary depending on pollinator groups, interventions, habitats, and spatio‐temporal conditions. In agreement with previous studies, pollinator groups responded differently to the interventions (Bihaly et al. 2024; Dainese et al. 2017). In urban areas, bumblebees and butterflies are the big'winner' (Theodorou et al. 2020), while other wild bees and hoverflies are also promoted by these actions. In contrast, domestic honey bees are not affected by these interventions in urban habitats, perhaps explained by their strong dependence on their hive locations. Although honey bees contribute to plant pollination, placing hives in cities is not a sustainable promoting approach for urban pollinators (Casanelles‐Abella and Moretti 2022; Ropars et al. 2019), as it increases resource competition and pathogen spillover for wild pollinators (Colla 2022). Contrary to Kennedy et al. (2013) and Shackelford et al. (2013), but in agreement with Zamorano et al. (2020), we found stronger effects on pollinator abundance than on species richness; see, for example, other wild bees and hoverflies. This probably reflects higher benefits for generalist over specialist species (van Klink et al. 2023), as well as the importance of the local species pool, which constrains species richness without limiting abundance (Pärtel et al. 1996). The urban‐filter effect may favour species adapted to high disturbance, habitat loss, fragmentation, and warmer temperatures, selecting against sensitive (e.g., oligolectic and kleptoparasitic bee) species, which are difficult to promote with these interventions (Buchholz and Egerer 2020; Dietzel et al. 2024; Fournier et al. 2020; Geppert et al. 2022; Venn et al. 2023). The neutral results obtained for non‐syrphid fly richness and flower‐visiting beetle abundance could be caused by their dependence on other resources (e.g., for larval stage (Cook et al. 2020; Gómez‐Martínez et al. 2022)). These resources were not considered here and the results of these two taxa were based on a few datasets, thus should be interpreted with caution.

To boost pollinator diversity, interventions typically focus on enhancing floral resources (Dietzel et al. 2023; Norton et al. 2019). Pollinators' occurrence and fitness are highly dependent on nectar and pollen quantity and quality, depending on the floral diversity (Szigeti et al. 2016; Vaudo et al. 2015). However, these floral rewards are not the only limiting factors; pollinators require a wide variety of resources for their larvae, nesting, or overwintering (Requier and Leonhardt 2020; Wood et al. 2015). Thus, any type of intervention can simultaneously have either positive or negative effects, depending on focal taxa resource needs or specific circumstances of habitats, making the generally positive picture more complex (Colla 2022). For instance, dense and tall vegetation that accompanies extensive mowing regimes may increase floral resources while decreasing the availability of underground nesting sites (Albrecht et al. 2023). Moreover, extensive mowing or abandonment were not even the most impactful intervention. Although flower sowing mostly had greater impacts than other interventions, it usually requires more effort and costs, making it less likely to be implemented over large areas (Schaub et al. 2021; Süle et al. 2023). Furthermore, habitat type had a major influence in several cases: the investigated variables generally responded better to interventions in road verges, whereas rarely in gardens, likely due to their greater pre‐existing diversity (Baldock et al. 2019), which is more difficult to enhance compared to more degraded road verges (Dietzel et al. 2023; Phillips et al. 2020). Pollinators in degraded habitats can be promoted more effectively (Tscharntke et al. 2005), while restoring slightly degraded ecosystems can be beneficial for the conservation of biodiversity (Kleijn et al. 2009), possibly also for urban habitats.

In our study, the sampling year and period, and age of intervention had small impacts. This result is in contrast to our knowledge about restoration outcomes and plant‐pollinator systems, which are strongly impacted by annual and seasonal weather fluctuations (Cane 2021; Herrera 2019; Rojas‐Botero et al. 2023). In addition, the impact of intervention's age due to succession (Albrecht et al. 2021; Buhk et al. 2018; Pywell et al. 2011) remains an unsubstantiated hypothesis here. This may be explained by the short‐term management and monitoring plans (Albrecht et al. 2020). Only a small portion of the datasets (< 30%) provided information on the intervention's age, and most of them covered either one (71%) or two years (21%), with only two datasets covering three years. However, achieving long‐term success should be the purpose of these interventions, which need follow‐up and sometimes additional management practices (Manninen et al. 2010), such as periodic overseeding and mosaic mowing (Neumüller et al. 2021; Parmentier 2023).

The site location seems to be an important factor influencing the effectiveness of interventions. The gathered studies originated from the whole continent. However, data coverage varied with some hotspots (e.g., Western countries), while other regions were data deficient (e.g., Mediterranean countries). Most of the significant treatment effects for honey bees were observed in Central Europe, probably due to the prevalence of beekeeping in this region (Potts, Roberts, et al. 2010). However, the similar spatial patterns for butterflies may have originated from their specific (larval and imago) resource requirements, preserved mostly in the more diverse habitats in Central Europe (Aguilera et al. 2019; Kőrösi et al. 2014). The continent‐scale differences in management actions, regulations, and biodiversity loss (Mainz and Wieden 2019; Török et al. 2020) may also underlie the above and some northwest‐southeast spatial patterns.

Taken together, the details behind the general positive roles of urban pollinator‐promoting interventions have only started to unfold, raising more research questions and innovation opportunities. To safeguard pollinators, implementations need co‐design processes with local stakeholders (Collins et al. 2024). During any steps forward, they need detailed reconsideration based on regional socio‐ecological characteristics (Kronenberg 2015; Southon et al. 2017), integrating wide‐scale, comprehensive overviews, guidelines, and recommendations (Baldock 2020; Millard et al. 2021; Tremblay and Underwood 2023; Wilk et al. 2019).

### 4.2 Limitations, Knowledge Gaps, and Perspectives

Promoting pollinators is receiving considerable public and scientific attention, while awareness and appropriate management approaches for urban green spaces are still emerging. As we move towards pollinator‐friendly cities (Connolly et al. 2018; Wilk et al. 2019), researchers, stakeholders, and citizens could all benefit from studies investigating these interventions (Beaurepaire et al. 2024; Southon et al. 2017; Süle et al. 2023), especially if they identify limitations. As an example, our European‐wide datasets are under‐represented in Mediterranean regions, which have several unique habitats of outstanding value for pollinator communities (Orr et al. 2021). Similarly, the majority of these pollinator promotions have concentrated on flower sowing and extensive mowing in parks and road verges, lacking some novel interventions and habitats. Furthermore, only a few studies recorded detailed background information besides the intervention and habitat types, such as vegetation parameters that could be proxies of floral resources and management intensity (Dylewski et al. 2019; Klein et al. 2020; Milberg et al. 2016). This lack, as well as limitations of the meta‐analytic approach in general, makes it difficult to reveal any non‐linear impacts, for example, the potential of the intermediate management intensity (Millard et al. 2021; Parmentier 2023; Rada et al. 2023). To develop and introduce innovative actions reaching higher naturalness and maintaining pollinator‐rich habitat fragments within cities, more comprehensive investigations are necessary involving under‐represented regions, habitats, interventions, pollinators, and resources. However, our synthesis could provide some recommendations for developing and implementing innovative measures (Table 2).

**TABLE 2:** Urban pollinator‐promoting interventions: Implementation, considerations (pro contra co‐benefits & trade‐offs), and recommendations.

Intervention type	Implementation	Pro (strengths)	Contra (threats)	Co‐benefits & trade‐offs	Recommendations
Abandonment & extensive mowing^i^ Ref: 1, 2, 3	Leaving green space undisturbed or extensively managed to regenerate in its natural way, mostly one late autumn or two mowings per year (similar to set‐aside or extensive grasslands in agricultural landscapes)	Higher vegetation^s^ producing flowers and seeds; Increasing plant species richness (i.e., floral and larval host plant resources)^s^; offering shelter and overwintering places; buffering micro‐climate	Full abandonment may reduce floral resources by succession	Preventing erosion; Might be cost‐effective; Potential enrichment of undesirable (e.g., tick, invasive plant) species; Without proper maintenance, sites could be ‘untidy’ due to garbage accumulation	Adjust the duration of abandonment, as well as the timing and intensity of mowing to specific regions and years; Avoid organic matter accumulation that facilitates grasses and shrubs; Monitoring of invasive plant species; Avoid introducing in crowded public space; Improve with overseeding and mosaic mowing
Flower sowing^i^ Ref: 3, 4, 5	Sowing seed mixtures in small patches and along linear (infra‐) structures (similar to agricultural flower strips)	Offer high amount of floral resources^s^; Outsanding local impact^s^; Potential hotspot for spreading beneficial native species	Native, local seed mixtures are scarcely available while the few widespread (non‐native) mixtures pose risks due to homogenisation, invasion, and functional resource limitation	Plant composition is determinable; Impressive aesthetic values for most citizens; Expensive due to sowing, watering, and weeding	Reveal soil characteristics and seed bank before establishment; Use seed mixture of native and mostly perennial plants with complementary floral traits; Maintain in medium/long‐term; Combine with mosaic mowing
Combined practices (to keep or reach higher biodiversity)^i^ Ref: 6, 7, 8	Enhance the naturalness of urban spaces by nature‐friendly approaches, e.g., reducing management intensity and chemical use, using mulch and peat, keeping dead wood	Flowering plants and beneficial (pollinator and biocontrol) insects can survive^s^	Pests also survive and may temporarily damage ornamental and cultivated plants	Cost‐effective; Relieving human environment from chemicals; Unusual, therefore ‘untidy’ areas might cause discomfort for citizens	Conduct comprehensive studies on continentand global‐scale social, economical, and ecological impacts; Harmonise sustainable approaches with citizens' priorities
Extensive grazing Ref: 9, 10, 11	Low intensity grazing (mostly by sheep and cattle) at larger urban grasslands, parks and orchards	Moderately and aggregately disturbed vegetation (by chewing and manuring) could be higher and produce more flowers and seeds that livestock disperse; Increasing plant species richness and heterogeneity	Over‐grazing can be destructive if stocking density is too high	Experience for kids; Produce meat; Probably cost‐effective; Citizens may be bothered by e.g., stench, excrement, coprophagous flies; Risk of infections and accidents; Incompatible at cities with high domestic pet populations; Majority of European cities lack urban shepherds and livestock infrastructure	Ensure the maintenance of good forage quality for the animals and avoid toxic species; Conduct studies on the implementation (e.g., required stocking densities), social engagement, and legislations from region to region
Bee hotels Ref: 12, 13, 14	Small, designed structures with holes made of reed, bamboo, wood, and brick	Beneficial for larvae of cavity‐nesting solitary bees and wasps, and overwintering species	Opportunity for generalist and invasive species, parasites, pathogens, and diseases	Spectacular, tiny elements of urban spaces where pollinators can be easily observed	Conduct studies on the role of pathogen spill‐over, competition between natives and non‐natives, landscape‐level effects, and multiplicative impacts
Bare ground surfaces & sand mounds Ref: 15, 16, 17	Creating small patches of bare soil	Beneficial for ground‐nesting species, excavating their own nests	Delayed impact: pollinator generations will increase several years after establishment; Poor management or abandonment threatens sites	Cost‐effective, e.g., small area is required; Unlikely to be accepted by citizens, and remain undisturbed by pets	Reveal soil characteristics and degradation stage before establishment; Studies and innovations are needed especially on interventions' size determination
Greening of architectural elements Ref: 18, 19, 20	Establishing and improving novel foraging and nesting places on roofs, balconies, terraces, and green walls	Diversifying and expanding the green infrastructure by involving architectural elements; May serve as stepping stones	Determined (mostly private) opportunities for establishment	Hard to evaluate its impact	Pay attention to low‐carbon transformation without damaging buildings; Innovations and (citizen science) studies are highly needed
Vegetation structure diversification Ref: 15, 21, 22	Providing diverse multi‐functional habitat containing larval host plants, shelter, shade, and sunbathing places, besides nectar and pollen resources	Offering diverse resources and complex habitats Enhancing functional diversity	Cities often lack the space and resources for large‐scale interventions	Integrative form of urban biodiversity initiatives, taking into account complex human‐nature systems	Consider avoiding invasive plants; Landscape‐level studies are needed

*Note:* The raised pro and con arguments are pollinator‐promoting and nature conservation‐focused, hand in hand with highlights on socio‐economic co‐benefits and trade‐offs. Recommendations call attention to knowledge gaps and offer actions to make specific interventions more favourable for pollinators and humans. The recommendations are based on our extensive but possibly incomplete expert knowledge. Therefore, each cell requires careful consideration during local interpretation and adaptation, taking into account potential regional differences. Superscripts indicate that: i = the intervention type is included in our synthesis by datasets; s = the specific statement is supported by our results. Reference studies where these interventions have already been investigated and insights have been provided: 1 (Garbuzov et al. 2015), 2 (Rada et al. 2023), 3 (Süle et al. 2023), 4 (Dietzel et al. 2023), 5 (Norton et al. 2019), 6 (Chalker‐Scott 2007), 7 (Muratet and Fontaine 2015), 8 (Shwartz et al. 2013), 9 (Abdulai et al. 2023), 10 (Davis 2021), 11 (Fekete et al. 2024), 12 (Geslin et al. 2020), 13 (MacIvor and Packer 2015), 14 (Rahimi et al. 2021), 15 (Baldock 2020), 16 (Fortel et al. 2016), 17 (Knapp et al. 2021), 18 (Benvenuti 2014), 19 (Braaker et al. 2017), 20 (Foster et al. 2017), 21 (Hausmann et al. 2016), 22 (Mach and Potter 2018).

As perspectives for pollinator‐friendly management, the mosaic mowing system by itself or in combination with overseeding would be a cost‐effective and sustainable way to provide season‐long food resources, climate‐adaptive vegetation, and even dynamic spaces for people, pets, and pollinators (Parmentier 2023; Unterweger et al. 2018; Wintergerst et al. 2021). In the case of extensive mowing, its timing and frequency (Kőrösi et al. 2014), while in the case of abandonment, the length of set‐aside (Mora et al. 2022) should be tested and adapted to local conditions. Extensive grazing also has the untapped potential to enhance pollinator habitats in urban areas (Davis 2021), but most cities currently do not permit or favour it, indicating a need for reconsideration. Similarly, urban buildings offer several greening opportunities, thus also pollinator‐promoting opportunities, such as balconies, green roofs, and also vertical surfaces like green walls (Braaker et al. 2017; Fox et al. 2022). Both cavity‐ and ground‐nesting insects could be promoted by wider opportunities, such as the introduced but understudied bee hotels (Rahimi et al. 2021), sand mounds, and bare soil surfaces (Baldock 2020; Potts et al. 2005), but note also their potential threats (Colla 2022; Fortel et al. 2016). Moreover, investigating the multiplicative effects of neighbouring interventions is a research gap, both in urban areas and agricultural landscapes (Garratt et al. 2023). Overall, urban pollinators require long‐term and large‐scale studies encompassing multiple taxa, intervention and habitat types (Baldock et al. 2019; Phillips et al. 2019; Theodorou et al. 2020). Future research should also address the traits and community composition of both plants and insects, as well as incorporate citizen science initiatives that actively engage urban inhabitants (Baldock et al. 2019; Griffiths‐Lee et al. 2023; Wei et al. 2016). Similarly, pros (e.g., more balanced microclimate, soil regeneration) and cons (e.g., maintenance costs, garbage accumulation), including citizens' perceptions (e.g., aesthetic co‐benefits vs. allergies and ticks as trade‐offs), need to be studied more (table 2; Cappellari et al. 2023; Geppert et al. 2024; Norton et al. 2019; Tremblay and Underwood 2023; Unterweger et al. 2017). Last but not least, given the emergence of novel urban habitats and substantial regional variation in anthropogenic environments (e.g., differences between public lands in the EU and the USA; Glenny et al. (2022)), there is a need to revisit and refine how these habitats are defined, considering in particular ecological functions and the incorporation of pollinator‐relevant criteria.

In the era of climate change and urban expansion, there are significant threats and opportunities that are leading to the enhancement of green spaces in urban settlements. In this context, anthropogenic habitats deserve research and restoration initiatives, without forgetting protected areas (Casanelles‐Abella et al. 2023; Chowdhury et al. 2023). To reach the ambitious goal of bringing people, plants, and pollinators together in multi‐functional, resilient, and sustainable infrastructures, citizens and stakeholders will need to develop locally adapted, collaborative, and research‐informed biodiversity initiatives and feedback systems.

## Author Contributions

G.S., A.B., and V.S. conceived of the presented idea and designed the study. G.S. and V.S. performed the data query. G.S. gathered the primary datasets provided by all authors. V.S. conducted data analyses with input and technical guidance from G.S., A.B., and D.K. G.S., A.B., and V.S. wrote the initial manuscript draft. All authors contributed data, to their interpretation, provided written feedback, and approved the final version.

## Peer Review

The peer review history for this article is available at https://www.webofscience.com/api/gateway/wos/peer‐review/10.1111/ele.70189.

## Supporting information


Figures S1‐S2:

Table S1:

Table S2:

Table S3:

Table S4:

Table S5:

Table S6:

## Data Availability

All data and code is publicly accessible at the Zenodo repository: https://zenodo.org/records/16037882 (DOI: 10.5281/zenodo.16037882).
