# Supplementary material for: Pollinator‐Promoting Interventions in European Urban Habitats—A Synthesis
Source: Ecol Lett. 2025 Aug 18;28(8):e70189. doi: 10.1111/ele.70189 (PMC12361876; doi:10.1111/ele.70189)
Supplement: Supplementary file 1 — Figures S1‐S2: [file ELE-28-0-s003.docx]

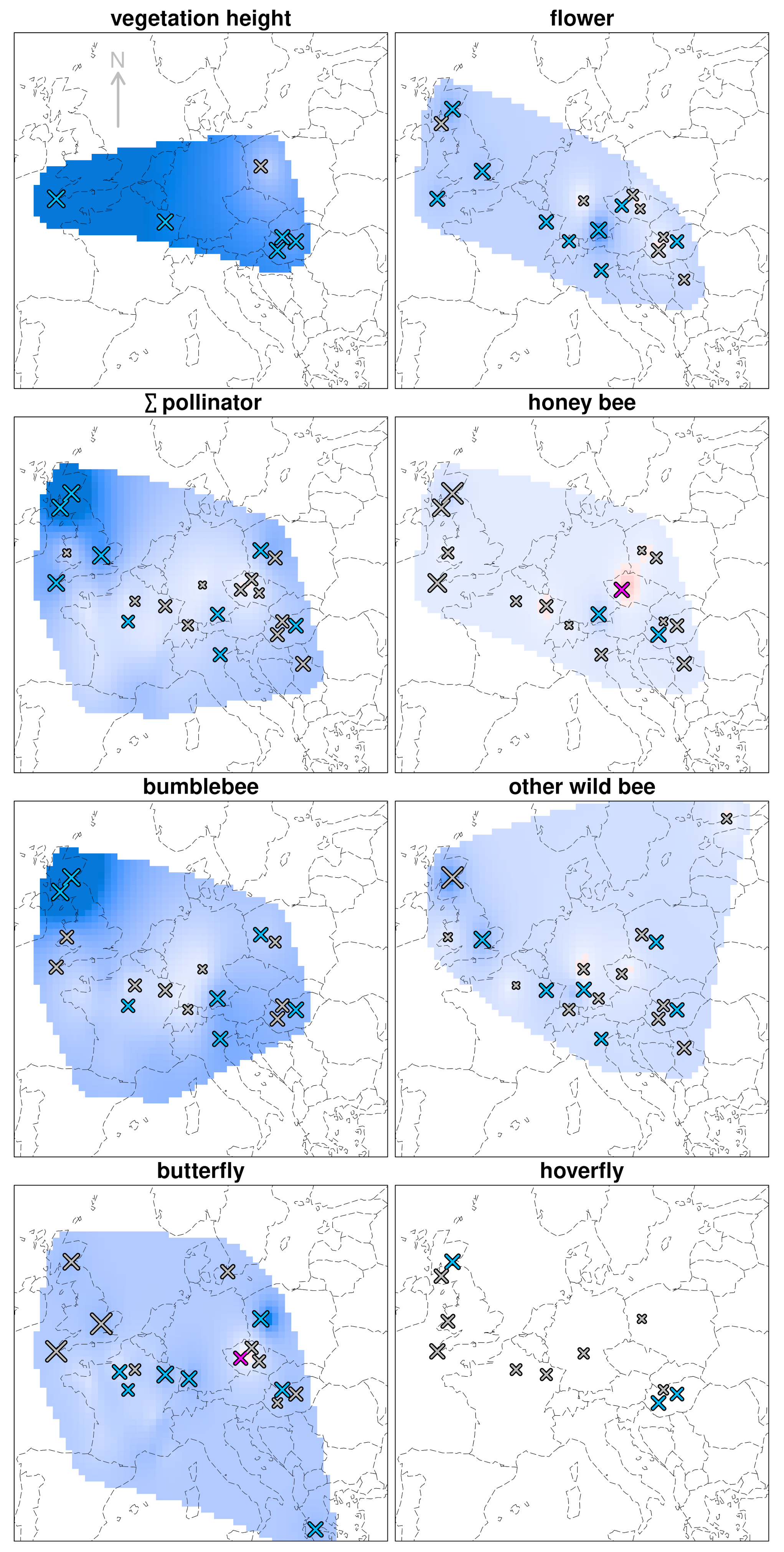


**Figure S1** Model predicted spatial patterns and study-level treatment effects on vegetation height, flower and pollinator abundances across Europe. Heat map represents the predicted spatial distribution from greater (darker) to lesser (lighter) positive (blue) and negative (magenta) treatment effects. Spatial distributions represent predictions on differences between treatments and controls only for models where AIC differences suggested spatial effects (Table S3). The prediction area covers the location of study sites with an additional 2° belt. Crosses represent study-level treatment effects, slightly jittered for visualisation. Grey crosses are non-significant, blue crosses are positive significant, while magenta are negative significant treatment effects from the study-level models. The size of crosses represents the model estimates (see details in Table S2).


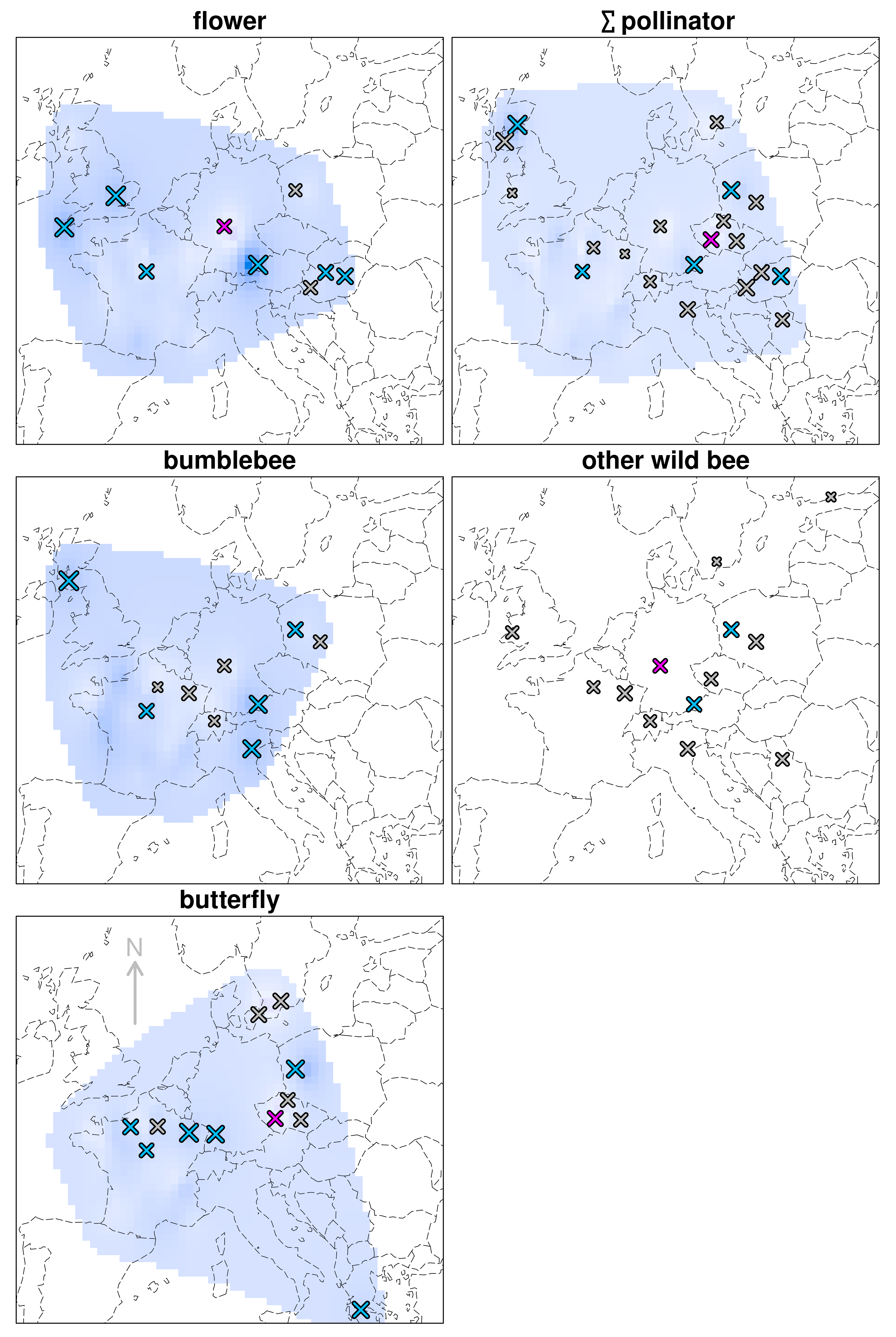


**Figure S2** Model predicted spatial patterns and study-level treatment effects on flower and pollinator species richness across Europe. Heat map represents the predicted spatial distribution from greater (darker) to lesser (lighter) positive (blue) and negative (magenta) treatment effects. Spatial distributions represent predictions on differences between treatments and controls only for models where AIC differences suggested spatial effects (Table S3). The prediction area covers the location of study sites with an additional 2° belt. Crosses represent study-level treatment effects, slightly jittered for visualisation. Grey crosses are non-significant, blue crosses are positive significant, while magenta are negative significant treatment effects from the study-level models. The size of crosses represents the model estimates (see details in Table S2).
